# A theoretical investigation of mixing thermodynamics, age-hardening potential, and electronic structure of ternary M^1^_1–*x*_M^2^_*x*_B_2_ alloys with AlB_2_ type structure

**DOI:** 10.1038/srep09888

**Published:** 2015-05-13

**Authors:** B. Alling, H. Högberg, R. Armiento, J. Rosen, L. Hultman

**Affiliations:** 1 Thin Film Physics, Department of Physics, Chemistry and Biology (IFM), Linköping University, SE-581 83 Linköping, Sweden; 2 Theoretical Physics, Department of Physics, Chemistry and Biology (IFM), Linköping University, SE-581 83 Linköping, Sweden

## Abstract

Transition metal diborides are ceramic materials with potential applications as hard protective thin films and electrical contact materials. We investigate the possibility to obtain age hardening through isostructural clustering, including spinodal decomposition, or ordering-induced precipitation in ternary diboride alloys. By means of first-principles mixing thermodynamics calculations, 45 ternary M^1^_1–x_M^2^_x_B_2_ alloys comprising M^*i*^B_2_ (M^*i*^ = Mg, Al, Sc, Y, Ti, Zr, Hf, V, Nb, Ta) with AlB_2_ type structure are studied. In particular Al_1–*x*_Ti_*x*_B_2_ is found to be of interest for coherent isostructural decomposition with a strong driving force for phase separation, while having almost concentration independent *a* and *c* lattice parameters. The results are explained by revealing the nature of the electronic structure in these alloys, and in particular, the origin of the pseudogap at *E*_*F*_ in TiB_2_, ZrB_2_, and HfB_2_.

Metal diborides constitute a large subgroup of the boride family crystalizing in several different structures. In particular those with the AlB_2_ type crystal structure are studied intesively for a wide range of properties: MgB_2_ demonstrates high-temperature superconductivity^1^,^2^, AlB_2_ is used for controlling solidification in Al metal casting^3^, and transition metal diborides, such as TiB_2_, ZrB_2_ and HfB_2_, combines high hardness, chemical and thermal stability with high electrical conductivity^4^,^5^,^6^,^7^,^8^. Also boron-richer metal borides, like the XYB_14_ phases^9^,^10^ as well as transition metal monoborides^11^,^12^ have demonstrated impressive mechanical properties in experimental and computational studies. These properties make the borides interesting candidates in the form of thin films for hard protective coatings for, e.g., cutting tools and for electrical contacts in demanding environments. However, their applications in industry have been hampered due to the technological challenge to synthesize boride thin films using physical vapor deposition^13^,^14^,^15^.

Despite the complexity involved, synthesis of thin films from diboride compound targets, such as TiB_2_, is at present industrially utilized through magnetron sputtering, with resulting hard or superhard TiB_2+*x*_, depending on degree of overstoichiometry^6^,^16^. Growth of superhard NbB_2−*x*_ thin films^17^, the possibility for epitaxial growth of ZrB_2_ as conductive contact layers on, e.g., SiC^18^, and the role of growth rate, residual gasses, and target purity for the film quality^19^ in magnetron sputtering of diborides has been discussed in the literature. Also, high power impulse magnetron sputtering of ZrB_2_^20^ has been performed and even arc-evaporation of TiB_2_ targets for the growth of boron-containing thin films have been demonstrated^13^,^21^.

As the field of thin film diborides for coatings applications is opening up, alloying is a natural next step for property enhancement. For Ti_1–*x*_Al_*x*_N hard coatings, isostructural clustering has been demonstrated as one of the reasons behind their technological success^22^,^23^, in a ceramics analogy of the age hardening of, e.g., AlCu alloys^24^. This raises the question if similar beneficial age-hardening phenomena are possible during heat treatment of diboride alloy films. Furthermore, only few studies have investigated the electronic structure of ternary diborides^25^ and there are outstanding questions also for binary diborides appreciating considerable theoretical investigations^26^,^27^,^28^.

Thus, a computational investigation which allows for an efficient and accurate derivation of alloy energetics^29^,^30^ and electronic structure is motivated to guide the emerging experimental efforts in this field. In this work we start by a first-principles scan of the mixing thermodynamics of all the 45 alloy systems formed by all 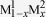
B_2_ combinations of the ten binary diborides MgB_2_, AlB_2_, ScB_2_, YB_2_, TiB_2_, ZrB_2_, HfB_2_, VB_2_, NbB_2_, and TaB_2_, all reported to crystalize in the AlB_2_ type structure. Several alloys, and in particular Al_1–*x*_Ti_*x*_B_2_, are identified as potential candidates for age-hardening due to isostructural decomposition tendency with limited lattice mismatch and are analyzed more in detail. Finally, the observed trends are explained by studies of the electronic structure of the alloys and how they change with composition. The findings are discussed in light of a here revealed modified explanation for the exisistance of a pseudogap at the Fermi level of TiB_2_: d-d electron interactions between transition metals in the simple hexagonal geometry.

## Results and Discussion

**Figure 1:**
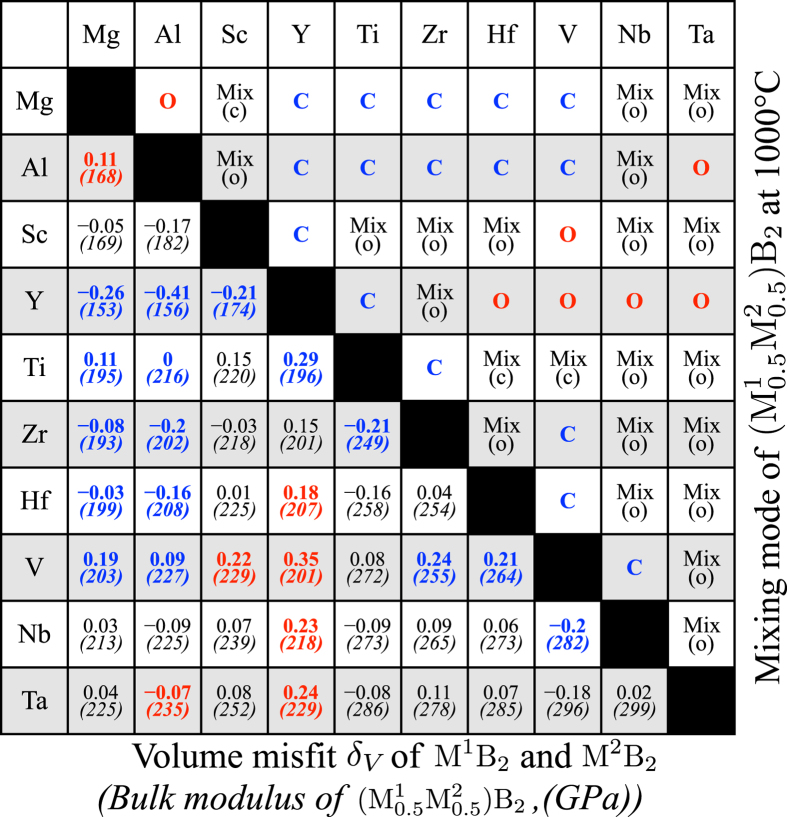
Upper-right: Overview of calculated mixing tendency in 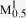

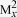
B_2_ systems at T = 1000 °C. “**C**” is clustering, “**O**” is ordering, “Mix(c)” is solid solution forming with clustering tendency at 0 K, “Mix(o)” is solid solution forming with ordered compounds stable at 0 K. Lower-left: Volume misfit *δ*_*V*_ of the pairs of binaries and the bulk modulus for the 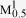

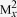

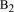
 ternary. Red and blue colors follow the ordering or clustering cases above for clarity.

As an illustrative example of a temperature where hard coatings are used, the mixing trends at 1000 °C are derived for all considered 45 ternary systems. The mixing trend for the composition 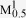

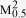
B_2_ is shown for each alloy in the upper right part of the matrix in Fig. 1.

Clustering, the formation of separate M^1^-rich and M^2^-rich regions, is found to be favored at 1000 °C in 16 cases marked with blue colored “C” in the upper right part of the matrix. Ordered 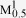

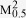
B_2_ phases are predicted to be lowest in free energy at 1000 °C for the seven cases marked with red colored “O”. Remaining 22 alloy systems are predicted to demonstrate solid solution miscibility at 1000 °C with either clustering or ordering tendencies at 0 K.

One common reason for lack of miscibility in isostructural alloys, according to the Hume-Rhothery rules, is lattice misfit. In the AlB_2_ type structure both *a*- and *c*-parameters should be considered, but as a first measure on lattice mismatch we study the volume misfit


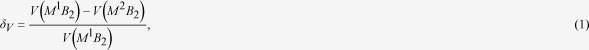


of the alloys. These values are presented in the lower left part of the Fig. 1 matrix. Also shown is the bulk modulus for the mixed 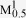

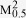
B_2_ compositions. It can be seen that all systems with a good solid solution formation ability has a limited volume mismatch of 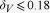. All alloys with larger mismatch favor either ordering or clustering.

**Figure 2:**
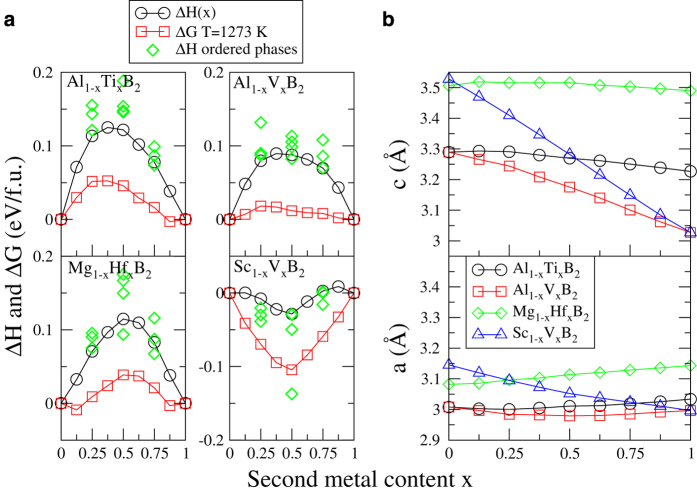
**a**) Mixing enthalpies of disordered and ordered alloys, as well as mean field free energy at 1000 °C for alloys that are identified as lattice matched clustering candidates, and an example of a ordering system. **b**) the *c*- and *a*- lattice parameters as a function of composition.

However, also lattice-matched systems can display clustering. Such alloys, with a small lattice mismatch, but a strong driving force for clustering are of particular interest for age-hardening potential. They are likely to form fully coherent interfaces and display spinodal decomposition with large composition fluctuations even when diffusion is limited, a concern for phase transitions in diborides^31^. The resulting nanostructure in the lattice can decrease dislocation mobility and increase hardness. For this reason, the alloys Al_1–*x*_Ti_*x*_B_2_, Al_1–*x*_V_*x*_B_2_, and Mg_1–*x*_Hf_*x*_B_2_ deserves further attention as they are predicted to display clustering and a small volume misfit as can be seen in the Fig. 1 matrix. Fig. 2(a) shows Δ*H* of ordered and disordered alloys for these system, as well as for the ordering Sc_1–*x*_V_*x*_B_2_ system for comparison, calculated with the 192-atoms SQS supercells for the disordered phases. Panel b) shows the *a*- and *c*-parameters as a function of composition for these systems.

Al_1–*x*_Ti_*x*_B_2_ is an almost perfectly lattice matched system, as can be seen in Fig. 2(b), but nevertheless displays a strong driving force for phase separation in terms of a positive mixing enthalpy and mixing free energy at 1000 °C, in particular in the AlB_2_-rich compositions. This is a very similar situation to the well studied Ti_1–*x*_Al_*x*_N alloys, where electronic structure effects have been found to be the origin of spinodal decomposition.^32^,^33^ The mixing enthalpies calculated for the disordered Al_1–*x*_Ti_*x*_B_2_ solid solutions have a maximum value of 0.125 eV/f.u. at 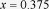. This is much lower than the values obtained by Zhang *et al*.^25^ who got 0.195 eV/f.u. using ordered structures, indicating the importance of a careful modeling of configurational disorder in this system. Our mixing enthalpy calculations strengthen the view of Ref 34 that, despite conflicting results, bulk experiments indicate lack of equilibrium miscibility of TiB_2_ and AlB_2_ within the temperature stability range of pure AlB_2_, *T *< 980 °C.

For Al_1–*x*_V_*x*_B_2_ alloys, the clustering driving force is weaker and the system is on the verge of mixing, while Mg_1–*x*_Hf_*x*_B_2_ is predicted to have a distinct clustering tendency for intermediate compositions, at 1000 °C. The Sc_1–*x*_V_*x*_B_2_ alloys on the other hand illustrate the mixing energetics of a system where a layered ScVB_4_ ordered structure is predicted to be stable even when configurational entropy of the disordered alloys are considered at 1000 °C, possibly demonstrating precipitation of ScVB_4_ in 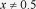 compositions.

**Figure 3:**
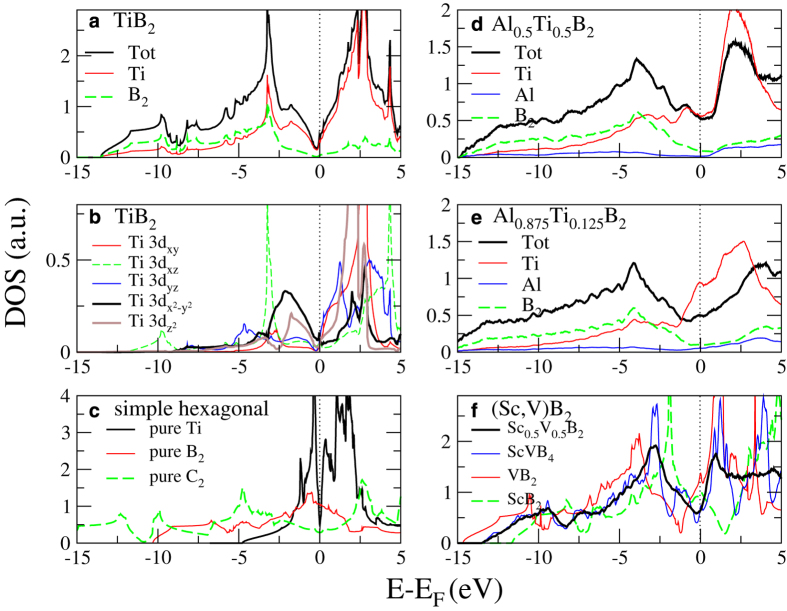
**a**) Total electronic DOS and **b**) symmetry projected Ti *d*-states of TiB_2_. **c**) Ti DOS of simple hexagonal structure without B, and B_2_, and C_2_ DOS without Ti. **d**) DOS of Al_0.5_Ti_0.5_B_2_, **e**) DOS of Al_0.875_Ti_0.125_B_2_, **f**) DOS of (Sc,V)B_2_ alloys.

The bonding physics behind the configurational tendencies of the alloys can be sought in their electronic density of states (DOS). The DOS of the illustrative case TiB_2_ is shown in Fig. 3(a). It displays the characteristic psuedogap at the Fermi level that has been debated in the literature and suggested to be due to the combination of B-B bonds, a charge transfer from the metal to the boron layer, and a strong bonding between B and Ti, seen in their common DOS peak around 3 eV below E_*F*_.^26^,^27^ However, less discussed^35^, one can also see that the DOS closest below and above E_*F*_ is dominated by Ti states with only a minimal inmixture of B character. Fig. 3(b) shows the symmetry projections of Ti 3*d* states demonstrating that the region between 2.5 and 0 eV below E_*F*_ is dominated by the orbitals of *e*_g_ symmetry, which extend in real space in the metal plane and orthogonal out of it in the direction of the metal atoms in the next metal plane above and below. On the other hand, the sharp peak at −3 eV is dominated by the *t*_2g_ orbital in the xz-direction, which in our unit cell set-up correspond to the direction of four of the nearest B neighbors.

To investigate if the pseudo gap could have an origin in the bonding of metal *d*-states as much as the metal-boron bondings, Fig. 3(c) shows pure Ti in the simple hexagonal structure resulting when removing B_2_ from the AlB_2_ structure unit cell. Also shown is the DOS of B_2_ when Ti is removed as well as C_2_ in the same geometry modeling the effect of two electrons transferred from each metal atom to the two boron atoms. Interestingly, it can be seen that a distinct pseudo gap at E_*F*_ appears also in the case of simple hexagonal Ti without boron. Almost precisely two out of four Ti valence electrons occupy the *d*-orbitals in this case, while the remaining valence electrons are of more delocalized character. Also for the C_2_ model, the *E*_*F*_ falls into a pseudo gap. Thus, we suggest that the distinct pseudo gap in transition metal diborides, present close to *E*_*F*_ for (Ti,Zr,Hf)B_2_, has its origin in a split of the *d*-orbitals caused by *d–d* interaction in the simple hexagonal geometry. This effect is then enhanced by charge transfer of electrons from the metal to boron as well as metal-boron bonding in the diborides.

Figure 3(d,e) show the effect of Al mixing into TiB_2_. While the B DOS is largely unchanged, the Ti partial DOS is changing. In particular, the peak corresponding to the 3*d–e*_g_ states just below E_*F*_ shifts towards higher energies as compared to pure TiB_2_. In Al_0.875_Ti_0.125_B_2_, this peak in the Ti partial DOS is right at E_*F*_. This can be understood as the *d–d* bonding, responsible for the pseudo gap formation in the Ti *d*-band, is disrupted by the introduction of Al, which *d*-orbitals are too high above the valence states in energy to take any part in the bonding.

One can also see that the sharp Ti peak 3 eV below E_*F*_ corresponding to Ti-B bonds is smeared out, possibly weakened, upon Al addition. These disturbances and breaking of *d–d* bonding in the electronic structure is the likely explanation for the large positive mixing enthalpy of Al_1–*x*_Ti_*x*_B_2_ and the driving force for clustering.

Finally, Fig. 3(e) shows that VB_2_ and ScB_2_ demonstrate more or less rigid band shifts as compared to TiB_2_. The Sc_0.5_V_0.5_B_2_ alloys, as expected, retain *E*_*F*_ in the pseudo gap, in a direct parallel to the composition which is energetically favored, seen in the corresponding panel of Fig. 2(a). Thus, alloying with higher or lower valency transition metals might be considered to increase the DOS at E_*F*_ of TiB_2_ and ZrB_2_ and thus the number of carriers in electrical contact applications. However, such attempts need to be balanced against disorder-induced scattering.

In conclusion, the mixing trends in 45 ternary diboride alloys of the AlB_2_ structure have been revealed. Metastable Al_1–*x*_Ti_*x*_B_2_ alloys are found to have a strong thermodynamic driving force for isostructural clustering combined with a small lattice mismatch and a high bulk modulus, which qualifies them for age hardening potential in thin film applications. The mixing trends can be understood from an analysis of the electronic structure and volume misfit revealed for the ternary diboride alloys. Metal *d–d* bonding is found to be part of the explanation for the pseudogap in the DOS of transition metal diborides.

## Methods

We use the density functional theory and the projector augmented-wave method^36^ as implemented in the Vienna Ab-Initio Simulation package (VASP)^37^,^38^ and the generalized gradient approximation as suggested by Perdew, Burke, and Ernzherhof for exchange-correlation energies^39^. For each considered structure the total energy of the calculation is converged to within 1 meV/atom with respect to the *k*-point sampling of the Brillouin zone. A plane-wave energy cut-off of 400 eV is used. Random 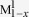

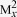
B_2_ alloys are modeled with the special quasi-random structure (SQS) method^40^ including 72 atoms per cell and the compositions *x* = 0.25, 0.50, and 0.75, in the initial scan and large 192 atom supercells with the compositions *x* = 0.125, 0.25, 0.375, 0.50, 0.625, 0.75, and 0.875 in the subsequent refined calculuations. Convergency tests show that the smaller cells give qualitative accurate representation of the mixing trends and converged equilibrium volumes, while the larger supercells are needed to obtain quantitative convergency of the mixing enthalpies.

The mixing enthalpies as a function of composition *x*, 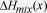, at zero pressure is calculated as


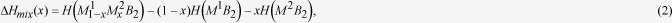


and for the configurationally disordered alloys the mixing free energy is approximated as 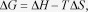 where 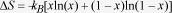 per formula unit (f.u.) is the configurational entropy of an ideal solution on the metal sublattice. Both sublattices are assumed to be stoichiometric.

In addition 10 ordered alloy structure with 24 atoms per cell at the compositions *x* = 0.25, 0.50, and 0.75 were considered for all systems. These were generated maximizing the numbers of M^1^-M^2^ bonds on the first two metal-sublattice coordination shells. The consideration of the pure binaries, the disordered SQS structures and the sample of ordered structures makes it possible to accurately scan the mixing trends in these ternary systems.

**Figure 4:**
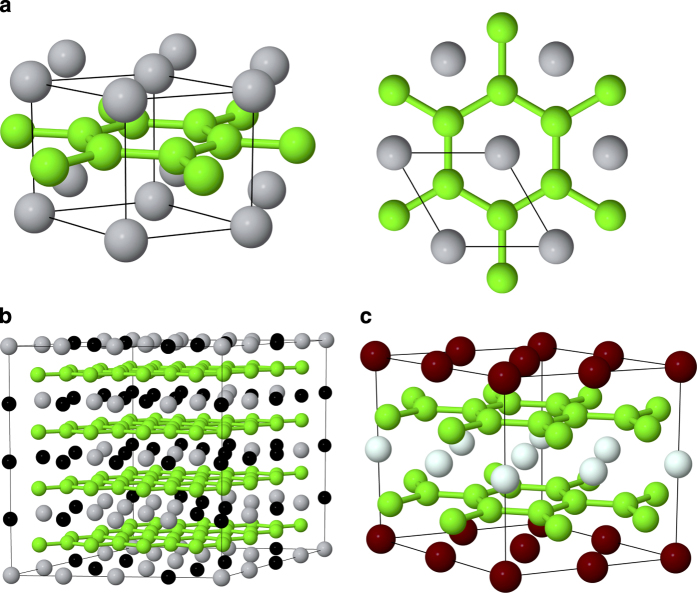
**a**) Side and top-view of the AlB_2_ crystal structure, **b**) a 192 atoms SQS supercell used for modeling e.g. Al_0.5_Ti_0.5_B_2_, **c**) the layered ordered alloy structure found to be lowest in energy for, e.g., ScVB_4_.

Figure 4 illustrates, using the Jmol package^41^, (a): the hexagonal AlB_2_ crystal structure, (b): a 192 atoms Al_0.5_Ti_0.5_B_2_ SQS supercell, and (c): the layered ordered configuration found to be the lowest energy structure for, among other systems, ScVB_4_. Boron atoms are shown in green, while metal atoms are shown in grey (Ti), black (Al), white (V), and dark red (Sc).

## Author Contributions

B.A. initiated the project. B.A. carried out the calculations. B.A., H.H., R.A., J.R. and L.H. contributed in the discussion and interpretation of the results. B.A. wrote the manuscript with input from H.H., R.A., J.R. and L.H.

## Additional Information

**How to cite this article**: Alling, B. *et al*. A theoretical investigation of mixing thermodynamics, age-hardening potential, and electronic structure of ternary M^1^_1-x_M^2^_x_B_2_ alloys with AlB_2_ type structure. *Sci. Rep*. **5**, 9888; doi: 10.1038/srep09888 (2015).
